# Comparative evaluation of estrus synchronization protocols on reproductive performance and estrus behavior in Barbados Black Belly sheep

**DOI:** 10.14202/vetworld.2023.2244-2249

**Published:** 2023-11-11

**Authors:** Krishna Mohan, Nitu Kumar

**Affiliations:** 1 Centre of Excellence on Indigenous Breed (CoEIB), RPCAU, Pusa, New Delhi, India; 2 TVO, Department of Animal Husbandry and Fisheries, Government of Bihar, India

**Keywords:** Barbados Black Belly, estrus behavior, estrus synchronization, ewes, Trinidad

## Abstract

**Background and Aim:**

Estrus synchronization of ewes has been accomplished using several protocols with various degrees of success in improving reproductive efficiency and obtaining the most effective protocol used in sheep farming. This study aimed to compare the effectiveness of three treatment protocols and to record the intensity and duration of estrus signs and pregnancy rate in Barbados Black Belly (BBB) sheep.

**Materials and Methods:**

Thirty-two primipara BBB ewes aged 18–24 months were equally divided into three treatment groups. T_1_: the ewes were injected intramuscularly with 2 mL Lutalyse (PGF_2α_) (10 mg) on days 0 and 10. T_2_: 1 mL Fertiline (50 μg; Gonadorelin acetate) on day 0 and 2 mL lutalyse (10 mg) on day 7. T_3_: 1 mL fertiline (50 μg) on day 0, 2 mL lutalyse (10 mg) on day 7, and 1 mL Fertiline (50 μg) on day 9. Estrus response was assessed using naturally mating rams and ewes. Pregnancy was determined using ultrasonography between 55 and 80 days after the last hormonal injection. The following estrus signs were noted: Swollen vulva, mucus discharge, sniffing, excitement, loss of appetite, mounting, and rapid tail movement.

**Results:**

Of the expressed signs, swollen vulva was most frequent, whereas loss of appetite and mucus discharge were the least overt signs recorded. The estrus response (%), onset (%), and duration (h) in ewe synchronization of the three treatment protocols were 100%, 58.3 ± 23.4%, and 48.0 ± 18.2 h (_T_1), 100%, 61.7 ± 41.2%, and 45.0 ± 27.0 h (T_2_), and 37.5%, 32.1 ± 1.7%, and 29.2 ± 1.25 h (T_3_), respectively. The pregnancy rates were 87.5%, 87.5%, 37.5%, and 50.0% in T_1_, T_2_, T_3_, and T_4_, respectively.

**Conclusion:**

Prostaglandin F_2α_+PGF_2α_ and GnRH+PGF_2α_ synchronization protocols were more effective in the fertilization of BBB ewes with better expression of estrus signs compared with the GnRH+PGF_2α_+GnRH (OVS) protocol.

## Introduction

The Barbados Black Belly (BBB) sheep is considered a very prolific or reproductively efficient breed that is well adapted to the tropical climate of the Caribbean. They are now widely considered a leading breed worldwide for their high prolificacy. Sheep breeding is a major source of income for farmers in developing countries. Given that improving reproductive performance can directly influence farmers’ income, using estrus synchronization protocols improves animal reproductive performance [1].

Reproductive efficiency is a critical trait that is regulated by various factors, such as environmental conditions, seasons, management systems, and nutrition [2]. Sheep are seasonal breeders and are actively productive during short daylight [3]. Therefore, assisted reproductive biotechnologies such as estrus synchronization could be used during the estrus and anestrus seasons to avoid genetic erosion and improve reproductive efficiency [4].

Several protocols have been used in small ruminants to induce and synchronize estrus and ovulation. Based on the role of prostaglandin (PG)F_2α_ in luteolysis of the corpus luteum (CL) [5], double PGF_2α_ injections are common for estrus synchronization in ewes. Given that PGF_2α_ efficacy is limited to the breeding season in which CL is active, different protocols using combinations of progesterone and gonadotropin-releasing hormone (GnRH) or human chorionic gonadotropin have been recommended for estrus synchronization outside the breeding season [6].

Sheep meat is consumed in the Caribbean community throughout the year, especially during celebrations, religious events, and tourist season. However, with the inability to satisfy local markets, the gap between the quality of commodities import and domestic production continues to widen, presenting potential investment opportunities. Therefore, investments in estrus synchronization practices can help this region overcome the cost of importing mutton, thereby moving closer to a more food-secure region.

Therefore, this study aimed to compare the effect of three synchronization protocols on estrus response, intensity, and duration and analyze the effectiveness of the protocols through pregnancy diagnosis using ultrasonography in BBB sheep.

## Materials and Methods

### Ethical approval

This study was approved by the Ethical Committee (No. CEC987/03/19) of the University of the West Indies (UWI).

### Study period and location

The study was conducted from August to December 2019 at the UWI field station animal farm. The climate of Trinidad and Tobago is tropical, with high relative humidity where the average minimum temperature ranges from 21.0°C to 23.0°C, and the average maximum temperature is 32.0–35.0°C and is situated between 10° 2’ and 11° 12’ N latitude and 60° 30’ and 61° 56’ W longitude.

### Animals

Thirty-two (n = 32) non-pregnant ewes (1.5–2.5 years of age, weighing 40–45 kg with body condition scores of 2 and 3) were selected from the University Field Station. The selected animals were examined for their health condition and other abnormalities through routine observation and examination using ultrasonography before the experiment started. All animals were free from anatomical and reproductive disorders and not health problems. Four (n = 4) healthy rams were also selected for mating.

### Animal housing and feeding

All animals were housed under a housing system with raised slatted floor pens (one pen per treatment group) and sufficient space for animal movement. The animals were fed as per the standard feeding practice used in the UWI field station farm with freshly cut forages and concentrates. In addition, fresh water and mineral lick (block) were available *ad libitum*.

### Synchronization protocols

All estrus synchronization protocols began on the same day, defined as day 0. Ewes (n = 32) were randomly allocated into four major groups (three treatments: T_1_, T_2_, T_3,_ and one control: T_4_). The ewes in each treatment group (n = 8) were marked with different colors. The synchronization protocols were used with slight modification, and estrus was synchronized with PGF_2α_ double dose (T_1_), GnRH−PGF_2α_ (T_2_), and ovsynch (OVS; T_3_) protocols, as described below.

### Prostaglandin F_2α_ double dose (T_1_)

On day 0, each ewe was intramuscularly injected with 10 mg (2 mL) PGF_2α_ (Lutalyse, Zoetis, USA). A second dose of PGF_2α_ (10 mg) was administered 9 days later. The ram was placed to identify ewes in heat and mate them for 7 days.

#### Gonadotropin-releasing hormone−PGF_2α_ (T_2_)

On day 0, each ewe was intramuscularly injected with 50 μg (1 mL) of GnRH analog (fertiline; gonadorelin acetate, Vetquinol, Canada), followed by PGF_2α_ lutalyse; dinoprost tromethamine, Zoetis, USA (10 mg) injection on day 7.

#### Gonadotropin-releasing hormone−PGF_2α_−GnRH (T_3_)

In this study, the OVS protocol was applied when an initial dose (50 μg) of GnRH analog (fertiline; gonadoreline acetate, Vetoquinol, Canada) was administered on day 0 to enable synchronized ovulation in ewes. The PGF_2α_ (10 mg; lutalyse) was intramuscularly administered 5 days later to remove the resulting CL. The second dose of GnRH analog (fertiline; gonadoreline acetate, Vetoquinol) (50 μg) was administered 2 days after PGF_2α_ (day 7) to increase ovulation synchrony.

#### Control group (T4)

Ewes (n = 8) were set aside as the control group, and they were not administered hormone injections.

### Estrus detection and mating

Estrus signs were observed through both direct visual observation and the use of a fertile ram after the application of the synchronization protocols. In the three treatment protocols, observation of estrus was initiated 12 h after the last injection. The occurrence of estrus in the ewes was monitored hourly by observing various behavioral estrus signs in addition to ram (teaser) parading, thrice daily for 30 min. Estrus observation using rams continued near ovulation time. The reaction of the treated ewes to the rams was noticed through sniffing, rapid tail movement, and finally standing to be mounted by the rams.

### Onset, intensity, and duration of estrus

Ewes were observed for estrus onset and duration for 72 h using visual observation and a teaser ram. Estrus onset was calculated in hours from the time of administration of the last hormonal injection to the time of the first appearance of estrus symptoms to the disappearance of estrus signs. Estrus intensity was measured using the method outlined by Mohan and Prakash [7].

The recorded parameters were as follows:

- The incidence of behavioral estrus signs (swollen vulva, mucus discharge, sniffing, excitement, loss of appetite, mounting, and rapid tail movement) after the onset of spontaneous estrus
- Comparison of reproductive performance between different synchronization protocols in terms of estrus response (%), onset (h), duration (h), and conception rate (%).

### Pregnancy diagnosis

Ultrasonography (Easi-scan ultrasound B-mode 5 MHz; transabdominal) was used to diagnose pregnant ewes between 55 and 80 days after the last hormonal injection.

### Statistical analysis

The collected data were analyzed using the Statistical Package for the Social Sciences (SPSS) version 23.0 (IBM SPSS, NY, USA). The mean and standard errors were calculated using GraphPad Prism 5.0 (GraphPad Software, San Diego, CA, USA). Estrus and pregnancy rates were compared using the Chi-square test.

## Results

### Effect of the synchronization protocol

Table-1 shows the comparison of reproductive performance, namely, onset of estrus, estrus response, duration of estrus, and conception rates between different synchronization protocols in BBB ewes.

**Table-1:** Comparison of reproductive performance between different synchronization protocols in Barbados Black Belly ewes.

Treatment group	No. of ewes	Estrus response (%)	Onset of estrus (h)*	Duration of estrus (h)	Conception rate (%)
PGF_2α_–PGF_2α_ (T1)	8	100^a^	58.3 ± 23.4^ab^ (32–82)	48.0 ± 18.2 (24–72)	87.5 (7/8)
GnRH–PGF_2α_ (T2)	8	100^a^	61.7 ± 41.2^ab^ (34–85)	45.0 ± 27.0 (22–70)	87.5 (7/8)
GnRH–PGF2_α_–GnRH (T3)	8	37.5^c^	32.1 ± 1.7^d^ (24–52)	29.2 ± 1.2 (18–38)	37.5 (3/8)
Control (T4)	8	50.0^b^	50.2 ± 3.1^bc^ (30–80)	24.4 ± 1.5 (12–36)	50.0 (4/8)
p-value	-	-	0.852	0.869	0.767

* Hours after the end of hormonal treatment. Means bearing at least one common superscript in a column did not differ statistically (p ≥ 0.05), otherwise significant at 5% level (p < 0.05), GnRH=Gonadotropin-releasing hormone, PGF_2α_=Prostaglandin F_2α_

### Estrus response

Estrus response was observed through direct visual observation and the use of fertile ram after applying the synchronization protocols. The obtained estrus response revealed that the estrus rate differed significantly (p < 0.05) in some of the treatment groups, where it was maximum (100%) with the PGF_2α_−PGF_2α_ (T_1_) and PGF_2α_−GnRH (T_2_) synchronization protocol (Table-1). The estrus response was lower (37.5%) in GnRH−PGF_2α_−GnRH (T_3_) and (50%) in the control (T_4_) group and differed significantly (p < 0.05) compared with T_1_ and T_2_ (Table-1).

### Onset of estrus

The onset of estrus was calculated in hours from the time of administering the last hormonal injection to the time of the first appearance of estrus symptoms and signs. Estrus responses were 58.3 ± 23.4, 61.7 ± 41.2, 32.1 ± 1.7, and 50.2 ± 3.1 in T_1_, T_2_, T_3_, and T_4_, respectively. Statistical significance (p < 0.05) was detected between the treatment groups (Table-1).

### Duration of estrus

The mean duration of estrus was 48.0 ± 18.2 h (24–72 h), 45.0 ± 27.0 h (22–70 h), 29. 2 ± 1.2 h (18–38 h), and 24.4 ± 1.5 h (12–36 h) in T_1_, T_2_, T_3_, and T_4_, respectively (Table-1).

### Conception rates

The conception rates differed significantly among the treatment groups. The highest pregnancy rates were recorded in T_1_ and T_2_, whereas the conception rates were lower in T_3_ (37.5%) and T_4_ (50.0%) (Table-1).

### Incidence and duration of behavioral estrus signs

The incidence of various behavioral estrus signs after induced estrus with different synchronization protocols is shown in Table-2. Swollen vulva and sniffing were the main estrus symptoms and were observed in all ewes; mounting and rapid tail movement were the next most exhibited symptoms observed in seven of the eight ewes in the T_1_ and T_2_. Excitement was a minor symptom also observed in ewes, whereas none of the ewes exhibited signs of mucus discharge or loss of appetite in any treatment group. All estrus signs exhibited were not very prominent with medium to low intensity. In addition, the numbers of estrus signs expressed per animal were medium (1.14) to low (0.86) (Table-2).

**Table-2:** Incidence of various behavioral estrus signs exhibited by Barbados Black Belly ewes during treatment with synchronization protocols.

Estrus signs	Treatment groups (32)^a^			

	Number of responders ewes (mean intensity^b^)			

	T1*	T2*	T3*	T4**
Swollen vulva	8 (++)	8 (++)	3 (+)	5 (++)
Mucus discharge	0	0	0	0
Sniffing	8 (++)	8 (++)	3 (++)	5 (+)
Excitement	5 (+)	6 (++)	2 (+)	4 (+)
Loss of appetite	0	0	0	0
Mounting	7 (+)	7 (+)	2 (+)	5 (+)
Rapid tail movement	6 (++)	7 (++)	3 (+)	4 (+)
Number of symptoms and average intensity of estrus signs observed/animals	4.8 (1.14)	5.1 (1.29)	1.9 (0.86)	3.3 (0.86)

a Indicates the total number of animals observed for estrus signs,

b On visual appraisal on a 3 point scale (+ low; ++ Medium; +++ high),

* Treatment groups: T1 (PGF_2α_−PGF_2α_); T2 (PGF_2α_−GnRH); T3 (GnRH−PGF_2α_−GnRH),

** Control group; T4. GnRH=Gonadotropin-releasing hormone, PGF_2α_=Prostaglandin F_2α_

Table-3 shows the duration of various behavioral estrus signs exhibited after the estrus synchronization protocol in BBB ewes. Swollen vulva was first observed at 58.1 ± 2.4 h (T_1_), 60.6 ± 3.1 h (T_2_), 33.3 ± 2.2 h (T_3_), and 0.67 ± 0.3 h (T_4_) and ended at 96.0 ± 1.6 h (T_1_), 93.0 ± 1.2 h (T_2_), 62.1 ± 1.8 h (T_3_), and 24.0 ± 1.1 h (T_4_). A swollen vulva was observed as a heat symptom in the treated ewes. Sniffing was initially observed in 8/8 ewes at 65.3 ± 3.1 h (T_1_), 63.5 ± 4.1 h (T_2_), 39.1 ± 2.8 h (T_3_), and 3.15 ± 2.8 h (T_4_) until after 86.0 ± 1.3 h (T_1_), 88.7 ± 2.4 h (T_2_), 59.3 ± 2.1 h (T_3_), and 21.9 ± 1.7 h (T_4_) after the synchronization protocols. Mounting was observed in 7/8 ewes at 69.6 ± 3.4 h (T_1_), 67.3 ± 3.4 h (T_2_), 70.1 ± 3.2 h (T_3_), and 09.2 ± 0.6 h (T_4_) and ended at 80.7 ± 2.3 h (T_1_), 81.5 ± 2.3 h (T_2_), 79.9 ± 1.3 h (T_3_), and 22.3 ± 0.3 h (T_4_).

**Table-3:** Duration of estrus signs (mean ± SEM) in Barbados Black Belly ewes (*n=*32) during treatment with synchronization protocols.

Treatment groups												

Estrus signs	T_1_*			T2*			T_3_*			T_4_**		
			
	Mean ± SEM (h)			Mean ± SEM (h)			Mean ± SEM (h)			Mean ± SEM (h)		
			
	Onset (h)	End (h)	Duration (h)	Onset (h)	End (h)	Duration (h)	Onset (h)	End (h)	Duration (h)	Onset (h)	End (h)	Duration (h)
Swollen vulva	58.1. ± 2.4	96.0 ± 1.6	37.9 ± 0.8	60.6 ± 3.1	93.0 ± 1.2	32.4 ± 1.9	33.3 ± 2.2	62.1 ± 1.8	28.8 ± 0.4	0.67 ± 0.3	24.0 ± 1.1	23.3 ± 0.8
Mucus discharge	0.0 ± 0.0	0.0 ± 0.0	0.0 ± 0.0	0.0 ± 0.0	0.0 ± 0.0	0.0 ± 0.0	0.0 ± 0.0	0.0 ± 0.0	0.0 ± 0.0	0.0 ± 0.0	0.0 ± 0.0	0.0 ± 0.0
Sniffing	65.3 ± 3.1	86.0 ± 1.3	20.7 ± 1.8	63.5 ± 4.1	88.7 ± 2.4	25.2 ± 1.7	39.1 ± 2.8	59.3 ± 2.1	20.2 ± 0.7	3.15 ± 2.8	21.9 ± 1.7	18.8 ± 1.1
Excitement	59.2 ± 2.8	69.3 ± 1.6	10.1 ± 1.2	60.1 ± 2.2	71.8 ± 1.3	11.7 ± 0.9	61.7 ± 3.4	69.3 ± 1.5	07.6 ± 1.9	1.15 ± 2.8	11.9 ± 1.7	10.8 ± 1.1
Loss of appetite	0.0 ± 0.0	0.0 ± 0.0	0.0 ± 0.0	0.0 ± 0.0	0.0 ± 0.0	0.0 ± 0.0	0.0 ± 0.0	0.0 ± 0.0	0.0 ± 0.0	0.0 ± 0.0	0.0 ± 0.0	0.0 ± 0.0
Mounting	69.6 ± 3.4	80.7 ± 2.3	11.1 ± 1.1	67.3 ± 3.4	81.5 ± 2.3	14.2 ± 1.1	70.1 ± 3.2	79.9 ± 1.3	09.8 ± 1.9	09.2 ± 0.6	22.3 ± 0.3	13.1 ± 0.3
Rapid tail movement	78.2 ± 1.6	87.7 ± 1.1	09.5 ± 0.5	77.8 ± 0.8	88.3 ± 0.6	10.5 ± 0.2	80.3 ± 1.6	89.4 ± 1.3	09.1 ± 0.3	13.3 ± 0.7	21.9 ± 0.3	08.6 ± 0.4

* Treatment group; T_1_ (PGF_2α_-PGF_2α_); T_2_ (PGF_2α_−GnRH); T_3_ (GnRH−PGF_2α_−GnRH),

** Control group; T_4_, GnRH=Gonadotropin-releasing hormone, PGF_2α_=Prostaglandin F_2α_, SEM=Standard error of the mean

The minor behavioral estrus signs observed after the synchronization protocols were excitement and rapid tail movement. The appearance of excitement started at 59.2 ± 2.8 h (T_1_), 60.1 ± 2.2 h (T_2_), 61.7 ± 3.4 h (T_3_), and 1.15 ± 2.8 h (T_4_), whereas mucus discharge and appetite loss were not observed in any ewe among the four treatment groups (Table-3).

## Discussion

In small ruminants, reproduction can be controlled using many recently developed synchronization methods. Estrus synchronization is a vital tool for successfully controlling reproduction efficiency, particularly conservation through population improvement [8]. It has been found that the estrus response ranges from 4.3% to 100%; however, it highly depends on the protocol used [9].

In our study, it is worth noting that all BBB ewes responded (100%) to double PGF_2α_ injection and PGF_2α_ with GnRH protocol with an 87.5% conception rate. The findings regarding estrus response in ewes are almost similar (90.9%, 93.7%, and 100%) [10]. In another study, Almadaly *et al*. [11] reported a 30% estrus rate with 100% pregnancy in Rahmani ewes using double PGF_2α_ injection protocol during the Mediterranean climate of northern Egypt. However, Fierro *et al*. [12] reported that PGF_2α_ based protocols generally achieve poor reproductive outcomes. The estrus response rate in Ghezel ewes following controlled internal drug release removal was 100%, with all ewes showing estrus over 3 days [13]. The use of two PGF injections ensures that the animal is synchronized because, depending on the stage of the estrus cycle, the first PGF injection may be ineffective. After the second injection, the ewes should show signs of estrus within 48 h. In another study, it was reported that GnRH stimulates the synthesis and secretion of gonadotropin hormone, follicle-stimulating hormone, and luteinizing hormone (LH). Gonadotropin-releasing hormone-PGF_2α_ combinations may enable the development of Graffian follicles earlier than the double PGF_2α_ regimen [10].

Herein, both GnRH and double PGF_2α_ injections gave a higher estrus response (100%) and conception rate (87.5%); however, GnRH−PGF_2α_−GnRH (OVS) results in a lower estrus response (37.5%) and conception rate (37.5%) in BBB ewes. Almadaly *et al*. [11] reported that the GnRH−PGF_2α_−GnRH (OVS) protocol could not improve fertility or even induce estrus during the non-breeding season in Rahmani ewes, which is consistent with our findings using GnRH−PGF_2α_−GnRH (OVS) in ewes. The lower estrus response and conception rate using the OVS protocol in the BBB ewes might be because of the dose and type of GnRH and PGF_2α_ analog used, as well as the season of the year. In addition, OVS-treated ewes may have previously experienced a false heat or expressed a complete estrous cycle before PGF_2α_ administration, according to Titi *et al*. [13]. In contrast, it has also been recently confirmed that the OVS protocol improved fertility in ewes and goats during the breeding season. Moreover, environmental conditions and seasons also influence estrus responses [14].

In this study, the duration of estrus was comparatively longer in T_1_ and T_2_ (48.0 h and 45.0 h), respectively, than in T_3_ and T_4_ (29.2 h and 24.4 h), respectively. Moreover, ewes treated with two PGF_2α_ injections showed the longest estrus duration (48.0 h), whereas the shortest was in the control group (24.4 h) (Table-1).

The results of this study agree with those recorded by Abu El-Ella *et al*. [14], where hormonal-treated ewes showed a higher duration of estrus (40.0–45.6 h) than the control group (24.0 h). Furthermore, Omontese *et al*. [15] reported similar findings in ewes. A comparative study on reproductive performance following estrus synchronization in South African indigenous sheep breeds was conducted, and it was reported that all Namaqua Afrikaner (100%) ewes responded to the synchronization protocols with the longest estrus duration (70.7 ± 7.2 h). However, the Namaqua Afrikaner sheep had the lowest rate of conception (44%). The longer duration of estrus in ewes may be due to elevated concentrations of circulating estrogen that ensure subsequent LH peak and thus increase the chance of a higher ovulation rate and successful fertilization. The surge in estrogen levels brings the animal into estrus and negatively affects progesterone levels. In another study, it was reported that a longer and varying duration of estrus may be due to higher estrogen levels in the blood, breed differences, age of the ewes, and geographical location [16].

Estrus response was observed through direct visual observation and the use of a fertile ram after applying the synchronization protocols. The obtained estrus response revealed that the estrus rate differed significantly (p < 0.05) in some of the treatment groups, where it was maximum (100%) with PGF_2α_−PGF_2α_ (T_1_) and PGF_2α_−GnRH (T_2_) synchronization protocols (Table-1). The estrus response was lower (37.5%) in GnRH−PGF_2α_−GnRH (T_3_) and (50%) in the control (T_4_) group and significantly different (p < 0.05) compared with T_1_ and T_2_ (Table-1).

Table-2 shows the incidence of different behavioral estrus signs after induced estrus with various synchronization protocols. The ewes exhibited estrus signs such as a swollen vulva and sniffing, which were noticed as main estrus symptoms. Mounting and rapid tail movement were the next most exhibited in seven of eight ewes in T_1_ and T_2_. Excitement was a minor symptom also observed in ewes, whereas none of the ewes showed signs of mucus discharge or loss of appetite in either of the treatment groups. The estrus signs exhibited were not very prominent with medium to low intensity. In addition, the numbers of estrus signs expressed per animal were medium (1.14) to low (0.86) (Table-2).

Table-3 shows the duration of various behavioral estrus signs exhibited after the estrus synchronization protocol in BBB ewes. Swollen vulva was first observed at 58.1 h (T_1_), 60.6 h (T_2_), 33.3 h (T_3_), and 0.67 h (T_4_) and ended at 96.0 h (T_1_), 93.0 h (T_2_), 62.1 h (T_3_), and 24.0 h (T_4_). A swollen vulva was observed as a heat symptom in the treated ewes. The minor behavioral estrus signs observed after the synchronization protocol were excitement and rapid tail movement, whereas mucus discharge and loss of appetite were not observed in any ewe among the treatment groups (Table-3).

The detection and expression of estrus in ewes are not as easily monitored if the ewes have been separated from the ram for some time. Ewes show less overt estrus behavior when they cannot hear, smell, or see the ram. Rapid tail movement or raised tail in the presence of a ram is a characteristic estrus behavior of the ewe. Other estrus behaviors, such as standing to be mounted by a ram or other ewes, are also typical of the ewes when experiencing estrus behavior but not as often as cattle. Secondary estrus behaviors such as nervousness, walking the fence, increased vocalizations for the ram, and decreased milk production and appetite were also observed in the ewes during estrus. In our finding, the estrus signs exhibited by ewes were not very prominent with medium to low intensity, and the numbers of estrus signs expressed per animal were medium (1.14) to low (0.86), which is almost similar to and supports the earlier reported studies.

## Conclusion

This study indicates that estrus synchronization with different protocols can be useful in improving the reproductive responses of BBB ewes, particularly estrus response, estrus intensity, and conception rate. The use of two PGF_2α_ and GnRH−PGF_2α_ injection protocols has been found to be effective in synchronizing estrus in ewes except GnRH−PGF_2α_−GnRH (OVS). However, further studies with more animal numbers are required to achieve better confirmatory findings.

## Authors’ Contributions

KM: Designed and performed the study, interpreted the data, and writing-original draft of the manuscript. NK: Data collection, editing, data analysis, and review of work. Both authors have read, reviewed, and approved the final manuscript.
